# Conditional deletion of KCC2 impairs synaptic plasticity and both spatial and nonspatial memory

**DOI:** 10.3389/fnmol.2023.1081657

**Published:** 2023-04-24

**Authors:** Anna Kreis, Farah Issa, Xavier Yerna, Caren Jabbour, Olivier Schakman, Marie de Clippele, Nicolas Tajeddine, Nathalie Pierrot, Jean-Noël Octave, Roberta Gualdani, Philippe Gailly

**Affiliations:** Institute of Neuroscience, Université catholique de Louvain, Brussels, Belgium

**Keywords:** GABA signaling, hippocampus, memory, chloride transporters, long term potentiation

## Abstract

The postsynaptic inhibition through GABA_A_ receptors (GABA_A_R) relies on two mechanisms, a shunting effect due to an increase in the postsynaptic membrane conductance and, in mature neurons, a hyperpolarization effect due to an entry of chloride into postsynaptic neurons. The second effect requires the action of the K^+^–Cl^−^ cotransporter KCC2 which extrudes Cl^−^ from the cell and maintains its cytosolic concentration very low. Neuronal chloride equilibrium seems to be dysregulated in several neurological and psychiatric conditions such as epilepsy, anxiety, schizophrenia, Down syndrome, or Alzheimer’s disease. In the present study, we used the KCC2 Cre-lox knockdown system to investigate the role of KCC2 in synaptic plasticity and memory formation in adult mice. Tamoxifen-induced conditional deletion of KCC2 in glutamatergic neurons of the forebrain was performed at 3  months of age and resulted in spatial and nonspatial learning impairment. On brain slices, the stimulation of Schaffer collaterals by a theta burst induced long-term potentiation (LTP). The lack of KCC2 did not affect potentiation of field excitatory postsynaptic potentials (fEPSP) measured in the stratum radiatum (dendrites) but increased population spike (PS) amplitudes measured in the CA1 somatic layer, suggesting a reinforcement of the EPSP-PS potentiation, i.e., an increased ability of EPSPs to generate action potentials. At the cellular level, KCC2 deletion induced a positive shift in the reversal potential of GABA_A_R-driven Cl^−^ currents (E_GABA_), suggesting an intracellular accumulation of chloride subsequent to the downregulation of KCC2. After treatment with bumetanide, an antagonist of the Na^+^-K^+^-Cl^−^ cotransporter NKCC1, spatial memory impairment, chloride accumulation, and EPSP-PS potentiation were rescued in mice lacking KCC2. The presented results emphasize the importance of chloride equilibrium and GABA-inhibiting ability in synaptic plasticity and memory formation.

## Introduction

Synaptic inhibition in the brain is largely mediated by the action of glycine and GABA acting on Cl^−^ permeable glycine receptors and GABA_A_ receptors (GABA_A_R) (Farrant and Kaila, 2007). The response to these agonists depends on the intracellular chloride concentration ([Cl^−^]_i_) which is tightly regulated by cation-chloride transporters. The Na^+^–K^+^–Cl^−^ cotransporter NKCC1 is responsible for Cl^−^ uptake accompanied by Na^+^ and K^+^ into cells (Gamba, 2005; Arroyo et al., 2013). It is highly expressed in the central nervous system (CNS) including the striatum, cortex, and hippocampus in both neurons and glial cells (Price et al., 2006; Tapia et al., 2019). The K^+^–Cl^−^ cotransporter KCC2 extrudes Cl^−^ from the cells. This isoform is neuron-specific (Payne et al., 1996). Interestingly, it undergoes a developmental upregulation during neuronal maturation (Rivera et al., 1999). The change of expression occurs just after birth (Rivera et al., 1999) and is responsible for the negative shift in the reversal potential of GABA_A_R-driven Cl^−^ currents (E_GABA_) that had been observed previously (Luhmann and Prince, 1991; Zhang et al., 1991). This shift changes GABAergic signaling from excitatory to inhibitory action (Chen et al., 1996; Ben-Ari et al., 2012). KCC2 is present in two major splice variants of the *Slc12a5* gene, KCC2a and KCC2b. Low expression levels of both variants can be detected in the pre-GABA shift. The expression of KCC2a remains low throughout life, while KCC2b expression undergoes developmental upregulation during the GABA shift, making it the predominant variant in the adult CNS (~90%) (Uvarov et al., 2007; Markkanen et al., 2014).

Alteration in KCC2 and NKCC1 equilibrium has been associated with a variety of neurological disorders. For example, in Down syndrome, the hippocampal expression of NKCC1 is increased, both in patients and in a mouse model of the disease (Deidda et al., 2015). In R6/2 mice, a Huntington’s disease mouse model, the expression of KCC2 has been reported to be decreased whereas NKCC1 expression increased (Dargaei et al., 2018). Several reports suggest that a change in the expression or in the activity of NKCC1/KCC2 might be responsible for some forms of epilepsy (for review, see (Fukuda and Watanabe, 2019; Liu et al., 2019)). In addition, it was shown that reducing KCC2 activity in the hippocampus was sufficient to induce temporal lobe epilepsy in mice (Kelley et al., 2018). KCC2 function could also be impaired in pain (Mapplebeck et al., 2019), schizophrenia (Arion and Lewis, 2011), Alzheimer’s disease (Doshina et al., 2017; Bie et al., 2022), and autism (Merner et al., 2015).

A decrease in the expression or in the function of KCC2 produces a change in E_GABA_, leading to a smaller GABA-induced hyperpolarization or even a depolarization. It therefore weakens the inhibitory action of GABA. This can be counteracted by enhancing KCC2 activity (Gagnon et al., 2013) or by inhibiting NKCC1. In some of the studies mentioned in the present article, pharmacological inhibition of NKCC1 with bumetanide indeed showed beneficial effects, including on cognitive symptoms (Deidda et al., 2015; Sivakumaran and Maguire, 2016; Dargaei et al., 2018).

GABAergic transmission is known to modulate hippocampal learning and memory (Andrews-Zwilling et al., 2012; Mohler and Rudolph, 2017; Sakimoto et al., 2021). So, in the present study, we investigated whether a decrease in KCC2 expression could by itself be responsible for cognitive impairments in adult mice. KCC2 knockout leads to respiratory impairment and perinatal death (Hubner et al., 2001; Dubois et al., 2018). Tornberg et al. therefore characterized the behavior of mice doubly heterozygous for KCC2 null and hypomorphic alleles that retain 15–20% of normal KCC2 protein levels in the brain. These mice presented an anxiety-like phenotype and were more susceptible to pentylenetetrazole-induced seizures. They also presented spatial learning deficits and reduced sensitivity to tactile and noxious thermal stimuli (Tornberg et al., 2005). However, it is difficult to discern whether the observed effects were directly due to a decrease in KCC2 expression and function or whether they were due to indirect effects on the development of the CNS, a process in which GABA polarity plays a crucial role (Fiumelli and Woodin, 2007; Peerboom and Wierenga, 2021).

Not only the expression but also the phosphorylation of KCC2 controls the timing of the postnatal E_GABA_ shift (Moore et al., 2019; Pisella et al., 2019). Delayed timing of the developmental onset of fast synaptic inhibition alters social interactions and memory retention. On the contrary, premature E_GABA_ hyperpolarization through KCC2 overexpression in cortical neurons impairs their morphological maturation (Cancedda et al., 2007) and results in a permanent decrease in excitatory synaptic signaling (Akerman and Cline, 2006).

In the study, we used a KCC2 CreERT2-lox system to knock down KCC2 at adult age. The mouse model expressed the Cre recombinase under the promoter of *CaMKIIα* gene, known to be expressed in several forebrain areas including the hippocampus (Madisen et al., 2010).

## Materials and methods

### Ethics statement

All animals were housed and handled according to the Belgian Council on Animal Care guidelines based on protocols approved by the Animal Ethics Committee of the Université catholique de Louvain (2016/UCL/MD/026 and 2020/UCL/MD/015). Animals were given access to food and water *ad libitum* unless otherwise stated. At appropriate experimental time points, all animals were humanely euthanized by an overdose of an anesthetic (5 mL kg^−1^ of a solution containing 67 mg mL^−1^ ketamine and 6.7 mg mL^−1^ xylazine) followed by decapitation.

### Animals

The generation of *KCC2^lox/lox^* mice was described previously (Seja et al., 2012; Godde et al., 2016). *KCC2^lox/lox^* mice were bred with Rosa26 Lox-Stop-Lox Tdtomato Reporter mice, (B6.Cg-Gt (Rosa)26Stortm14 (CAG-tdTomato)Hze/J, Jackson Laboratory) to generate the respective genotypes used for the experiments. They were then crossed with *CaMKIIα-CreERT2* transgenic mice expressing a tamoxifen-inducible Cre recombinase under the control of the mouse *CaMKIIα* (calcium/calmodulin-dependent protein kinase II alpha) promoter region (*B6; 129S6-Tg (CaMKIIα-Cre/ERT2*)1Aibs/J, Jackson Laboratory). Transgenic mice *KCC2^lox/lox^ CaMKIICre^+/−^* and their *KCC2^lox/lox^ CaMKIICre^−/−^* littermates were identified by PCR genotyping on genomic tail DNA. For KCC2, the oligonucleotide primers contained a forward 5′ CAACCTGAACTCCCAAGGATACCC 3′ and a 5′ TCTGCCTGGAACACTCTCCTGC 3′ reverse sequence. The reactions generated fragments of 300 bp for wild-type and 500 bp for the KCC2 floxed allele, respectively. For CaMKIICre ERT2, the primers for the triplex reaction consisted of ERT2.1 (sense) 5’ GGTTCTCCGTTTGCACTCAGGA 3′, ERT2.2 (antisense) 5’ CTGCATGCACGGGAC AGCTCT 3′, and ERT2.3 (antisense) 5’ GCTTGCAGGTACAGGAGGTAGT 3′. The reactions generated fragments of 290 bp for wild-type and 375 bp for CaMKIICre ERT2, respectively. For the immunofluorescent marker rosaTomato, the primers consisted of a 5’ AAGGGAGCTGCAGTGGAGTA 3′ forward and a5’ CCGAAAATCTGTGGGAAGTC 3′ reverse sequence for wild type as well as

5’ GGCATTAAAGCAGCGTATCC 3′ forward and 5’ CTGTTCCTGTACGGCATGG 3′ reverse sequence for rosaTomato. The reactions yielded fragments of 297 bp for wild-type and 196 bp for rosaTomato, respectively.

Only male mice at the age of 3 months were used in this study. Experiments were conducted during the 2nd and 3rd week post-tamoxifen-treatment, and mice were euthanized at the end of the third week after tamoxifen treatment.

### Behavior

#### Open field

The open-field test was used to assess non-forced ambulation as mice could move freely without any influence from the examiner. Briefly, mice were placed in a square arena (60 × 60 cm), and the video was tracked (Ethovision 6.1, Noldus; Wageningen, The Netherlands) for 20 min. The total distance covered by the animals and the time spent in the center vs. periphery were measured.

#### Morris water maze

The Morris water maze (MWM) test was used to assess spatial learning and memory (Morris, 1984). The maze consisted of a round pool with a diameter of 113 cm virtually divided into four quadrants (north, south, west, and east) filled with 26°C opaque water. Visual cues were placed around the pool. The platform was located at the center of the north-east quadrant. On the 1st day of training, mice learned to find the visible dark platform, and if they are not able to find the platform during a time span of 1 min, they were placed on the top of the platform. On the 2nd, 3rd, and 4th experimental day, the dark visible platform was replaced by an invisible see-through one. Animals were introduced to the MWM from different quadrants with 1 min time to find the invisible platform. On the 5th day, the animals were submitted to a probe test that allowed the animals to swim freely for 1 min in the pool without the escape platform. During the trial and the probe test, mice were video tracked. The time latency to reach the platform, the swim speed, and the time spent in each quadrant were measured (Boucherie et al., 2018).

#### Novel object recognition

The novel object recognition test was used to assess recognition memory. On the 1st day (training phase), mice were placed in a square arena (40 × 40 × 35 cm) containing two identical novel objects (two small bottles) and allowed to explore them for 10 min. On the 2nd day (test phase), the mice were placed in the same arena where one of the two original objects was replaced by another of different shapes and colors (a plastic cube) and were allowed to explore them for 10 min. During both phases, the mice were video-tracked (Ethovision 6.1, Noldus; Wageningen, The Netherlands). The exploration time of each object was measured, and a discrimination index (time spent exploring the novel object minus time spent exploring the familiar object/total time to explore both objects) was calculated.

#### Elevated plus maze

The elevated plus maze test was used to assess anxiety. Mice were placed in an elevated plus maze consisting of two opposing open arms (exposed place) and two opposing closed arms (safer place). Time spent in each arm and distance covered was recorded by a video tracking system (Ethovision) for 5 min.

#### Rearing and grooming

These tests were conducted in transparent plastic cages. After a period of 10 min habituation, the duration of grooming bouts and the number of rearings were recorded manually for 10 min.

#### Western blotting

Mice were sacrificed by anesthetics overdose given by intraperitoneal (IP) injection. Brains were dissected and the mouse hippocampi were taken, snap frozen in liquid nitrogen, and lysed in RIPA buffer (25 mM Tris HCl pH 7.6, 150 mM NaCl, 1% NP-40, 1% sodium deoxycholate, and 0.1% SDS) for 2 h at 4°C. Lysates were centrifuged at 4°C, 10,000 g for 5 min, and supernatants were kept at −80°C until use. For immunochemistry in the CA1 region, 350 μm-thick hippocampal sections were prepared (see below), and the CA1 region was cut out and treated as whole hippocampi. Protein concentration was determined by the bicinchoninic acid protein assay kit (BCA, Pierce), and the absorbance was subsequently measured with a NanoDrop spectrophotometer. Samples were heated for 15 min at 70°C. In the study, 10 mg of protein for each sample was loaded on a 10% SDS-polyacrylamide gel and transferred to a nitrocellulose membrane (Biorad). After blocking with 5% non-fat milk for 1 h, at room temperature, membranes were incubated in appropriate primary antibodies (KCC2 1:3000 (Merck 07–432), NKCC1 1:1000 (Abcam ab59791), β-tubulin 1:10000 (Neuromics MO1503), at 4°C, overnight. Membranes were then incubated with secondary antibodies coupled to peroxidase (Dako), and peroxidase was detected with Pierce ECL Plus (Thermo Scientific) on ECL hyperfilm (GE Healthcare). Quantification of protein levels was performed by densitometry and reported to β-tubulin expression.

### Histology

Experimental mice were deeply anesthetized by intraperitoneal injection (5 mL kg^−1^ of a solution containing 67 mg mL^−1^ ketamine and 6.7 mg mL^−1^ xylazine) of a solution containing 10 mg mL^−1^ ketamine and 1 mg mL^−1^xylazine. After intracardiac perfusion with a 4°C buffered 4% paraformaldehyde phosphate-buffered saline (PBS) solution, the brain was removed and postfixed in the same solution for 2 h at 4°C. Before freezing, brains were moved to a 20% sucrose solution and kept at 4°C until the tissue sank to the bottom of the tube. Whole brains were embedded in the optimal cutting temperature compound (Tissue-Tek OCT compound, VWR, Leuven, Belgium), frozen in isopentane cooled at −50°C, and stored at −80°C until use. Parasagittal 20 μm thick cryosections were cut and stored at −80°C. Brain sections were defrosted for 2 min at room temperature and then rehydrated with PBS washing buffer for 10 min at room temperature. Slices were then mounted with the Prolong gold antifade reagent with the DAPI mounting medium (Invitrogen). All brain sections were analyzed with the Apotome Z1 imager (Zeiss).

### Electrophysiology

#### Brain slice preparation

Animals were sacrificed by cervical dislocation, and their brains were harvested and transferred to ice-cold artificial cerebrospinal fluid (ACSF) composed of 126 mM NaCl, 3 mM KCl, 2.4 mM CaCl_2_, 1.3 mM MgCl_2_, 1.24 mM NaH_2_PO_4_, 26 mM NaHCO_3_, and 10 mM glucose (bubbled with 95%O_2_–5% CO_2_%). After the removal of the cerebellum and frontal cortex, brains were mounted onto a vibratome, and sagittal sections of 350 μm were cut in ice-cold ACSF to obtain the dorsal hippocampus. Brain slices were acclimatized in oxygenated ACSF at 32°C for at least 1 h before use.

#### Field potential recordings

Brain slices were transferred to the recording chamber while continuously being perfused by oxygenated ACSF (2 mL/min) at 30°C. Field excitatory postsynaptic potentials (EPSPs) and population spikes (PS) were evoked through a bipolar stimulating electrode which was placed in the Schaffer collaterals (SC) and recorded by AxoClamp 2B amplifier through a glass electrode which was back-filled with 2 M NaCl and placed in the CA1 region (the stratum radiatum and the stratum pyramidale respectively, measurements done in parallel, on different slices) (Lepannetier et al., 2018; Yerna et al., 2020). Stimuli consisted of 100 μs pulses of constant currents with intensity adjusted to produce 35% of the maximum response every minute. Responses were stabilized for 30 min (1 stimulation/min) after placement of the electrodes. All responses were digitalized using Digidata 1322A (Axon Instruments, United States) and recorded on a computer using WinLTP software (Anderson and Collingridge, 2007). LTP was induced by applying theta burst stimulus (TBS) consisting of five trains of four pulses (100 Hz) with 10 ms interval and 200 ms interval between each of the five trains (Wang et al., 2006). EPSP and PS responses were normalized to the pre-TBS baseline and defined as 100%.

#### Primary hippocampal cell culture preparation

*Cre^+^* and *Cre^−^* mouse pups (0-1P) were sacrificed, and the hippocampi were taken for primary cell culture. The hippocampal tissue was dissociated with a sterilized glass pipette in ice-cold FBS, and after 2 min, the cell debris was allowed to settle, and the cell supernatant was transferred into ice-cold BSA. After centrifugation (5 min, 4°C, 1000 RPM) the pelleted cells were harvested and transferred into the 37°C warm neurobasal medium. Neurons were plated on Poly-D-lysine coated glass coverslips (one hippocampus/coverslip) in a 24-well plate. Each culture (from the hippocampi of one pup) was genotyped. After 1 week, 2 mM hydroxy-tamoxifen was added daily to the cell culture over a period of 1 week (1 μmol/well). After 24 h of the last dose of hydroxy-tamoxifen, cells were patched using a gramicidin perforated patch clamp.

#### Patch clamp recordings

Patch-clamp recordings of hippocampal neurons were carried out at RT, using an EPC-9 amplifier controlled by Patch Master software. The resting membrane potential of neurons was measured in a whole-cell configuration using the following extracellular composition (in mM): 140 NaCl, 6 KCl, 2 CaCl_2_, 1 MgCl_2_, and 10 HEPES adjusted to pH 7.4 with NaOH. The intracellular solution had the following composition (in mM): 137.5 K-gluconate, 11 NaCl, 11 EGTA, 11 HEPES, and 11 glucose adjusted to pH 7.2 with KOH (310 ± 5 mOsm). The perforated patch clamp experiments (Kyrozis and Reichling, 1995) were performed using the following extracellular solution (in mM): 140 NaCl, 3 KCl, 3 CaCl_2_, 2 MgCl_2_, 10 glucose, and 10 HEPES, at a pH of 7.4. The pipette solution had the following composition (in mM): 150 KCl and 10 HEPES, at a pH of 7.4. All perforated patch clamp experiments were performed in the presence of 10 μM 6-cyano-7-nitroquinoxaline-2,3-dione disodium (CNQX), 10 M D-(−)-2-Amino-5-phosphonopentanoic acid (D-AP5) and 100 nM CGP 55845 to block AMPA, NMDA, and GABA_B_ receptors, as well as 1 μM tetrodotoxin (TTX) to block sodium channels. GABA was dissolved in the extracellular solution (100 μM) and applied through a patch pipette close to the cell body of the recorded neuron, using 10 ms pressure puffs (Picospritzer). Gramicidin (dissolved in DMSO) was added to a filtered pipette solution to obtain a final concentration of 80 μg/mL and sonicated 2 times for 10 s with 10 s interval. High-resistance cell-attached seals were obtained through the application of negative pressure. Changes in series resistance due to gramicidin pore formation were monitored by delivering 10 mV voltage steps. Appropriate pore formation was indicated by series resistance values of 50–100 MΩ. An AgCl wire was used as a reference electrode. The patch pipettes were pulled with a resistance of 4–7 MΩ using a DMZ-Universal puller. Currents were sampled at 10 kHz and digitally filtered at 2.9 kHz.

### Statistical analysis

Statistical analysis of all data points collected in experiments mentioned in this study was performed using GraphPad Prism 9. For each set of experiments, “*n*” represents one brain slice (LTP, histology), one animal (behavior, Western blot), or one neuron (patch clamp experiments). For all experiments except Western blots, the normal distribution of data points was assessed using the D’Agostino-Pearson omnibus normality test. For the comparison of two groups with normal distributions and equal variances, the two-tailed unpaired *t*-test was performed while for the comparison of two groups with unequal variances a two-tailed unpaired *t*-test with Welch correction was applied. Multiple comparisons were performed using ANOVAs with Holm-Sidak or Bonferroni *post-hoc* tests. For Western blot observations and grooming and rearing behaviors, the comparisons were done using a Mann–Whitney test (nonparametric). All experimental datasets are expressed as a mean ± standard error of mean (SEM) except Western blot data and for grooming and rearing behaviors, which are expressed as medians, 1st and 3rd quartiles, and 5th/95th percentiles (box plots). Statistical significance was fixed to a *p*-value of ≤0.05 (^*^p ≤ 0.05, **p ≤ 0.01, ***p ≤ 0.001).

## Results

### Conditional deletion of KCC2

**Figure 1:**
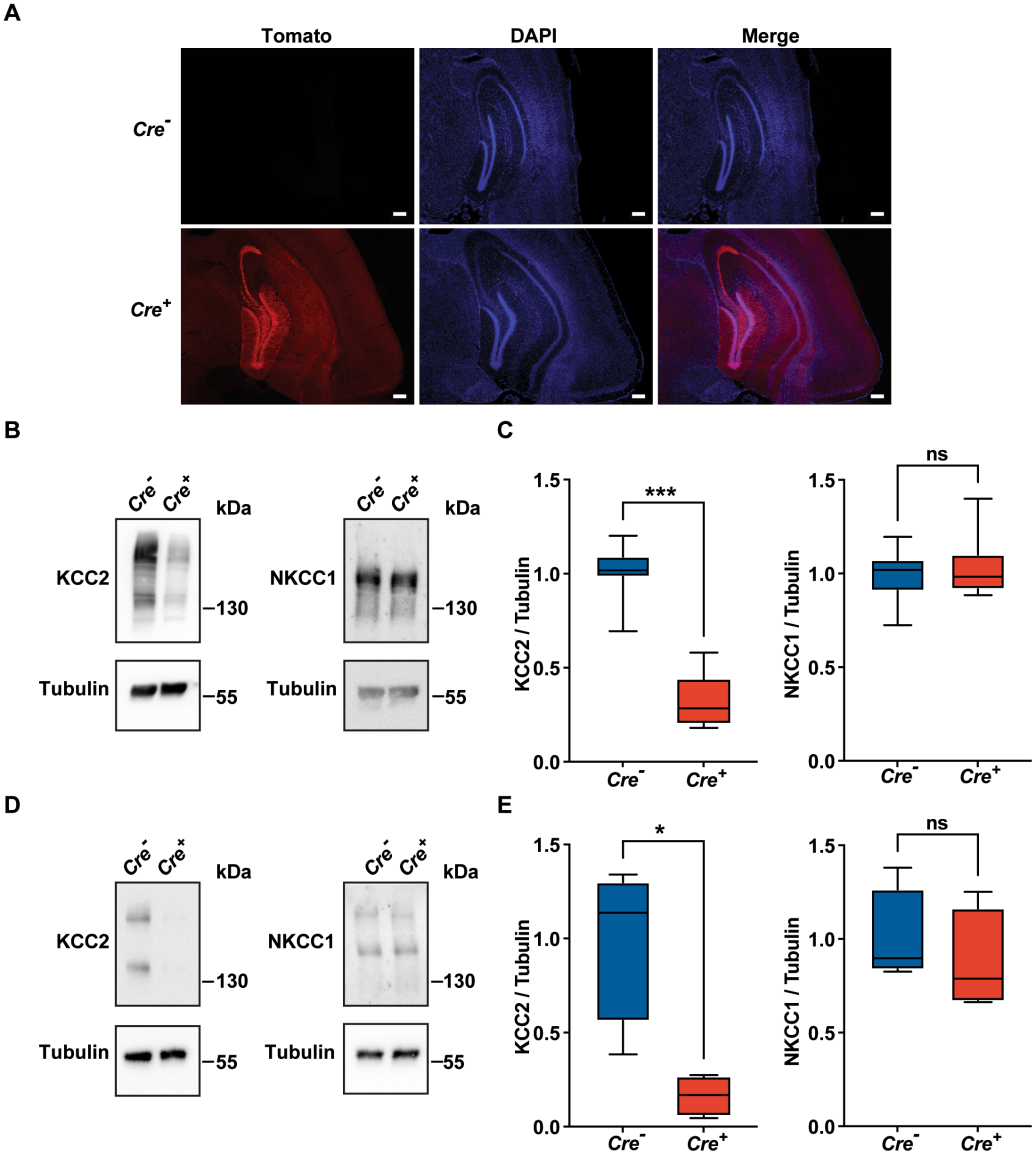
Validation of the *KCC2^lox/lox^ CamKIIα-CreERT2* mouse model. **(A)** Neuronal expression of rosaTomato in the cortex and hippocampus after tamoxifen treatment with DAPI counterstaining in *Cre^+^* and *Cre-genotypes*. Scale bar: 200  μm. **(B-E)** Quantification and representative Western blots of KCC2 and NKCC1 protein levels of whole hippocampi tissue lysates (*n* = 7; **(B,C)** and of CA1 regions lysates (*n* = 4; **D,E)** of 3  month-old animals. Protein amount was normalized to β-tubulin and expressed as the proportion of the mean KCC2/*β*-tubulin ratio of *Cre-samples*. (^*^p ≤ 0.05; ^***^p ≤ 0.001; Mann–Whitney test).

Homozygous KCC2^−/−^ mice die at birth probably due to a respiratory defect (Hubner et al., 2001). To avoid this perinatal lethality, we used KCC2 conditional knockout mice carrying floxed *KCC2* exons 2–5 (*KCC2^lox/lox^* mice) (Seja et al., 2012) and a *Rosa tdTomato* reporter gene. In order to achieve temporally controlled somatic mutagenesis specifically in neurons of the forebrain region, these mice were crossed with mice expressing the CreERT2 fusion protein under the control of the regulatory elements of the *CaMKIIα* gene (*CaMKII-CreERT2* transgene) (Erdmann et al., 2007; Schonig et al., 2012). Transgenic *KCC2^lox/lox^ CaMKIICre^−/−^* mice (hereinafter referred as *Cre^−^*) and their littermates *KCC2^lox/lox^ CaMKIICre^+/−^* (hereinafter referred as *Cre^+^*) were injected intraperitoneally once a day for 5 days with 1 mg of tamoxifen. After a delay of 10 days, the activation of the Cre recombinase was evident due to the expression of the fluorescent marker rosaTomato, especially in the cortex and in the hippocampus (Figure 1A), and we observed that the expression of KCC2 measured by Western blot was diminished by nearly 70% in whole hippocampus tissue lysates of 3-month-old *Cre^+^* animals compared to *Cre^−^* (Figures 1B,C). The quantification of NKCC1 protein levels in hippocampal tissue lysates did not show any variation between *Cre^+^* and *Cre^−^* mice. Similar observations were made specifically in the CA1 region, with a decrease of 84% in the expression of KCC2 with no effect on the expression of NKCC1 (Figures 1D,E).

### KCC2 deletion leads to cognitive impairment

**Figure 2:**
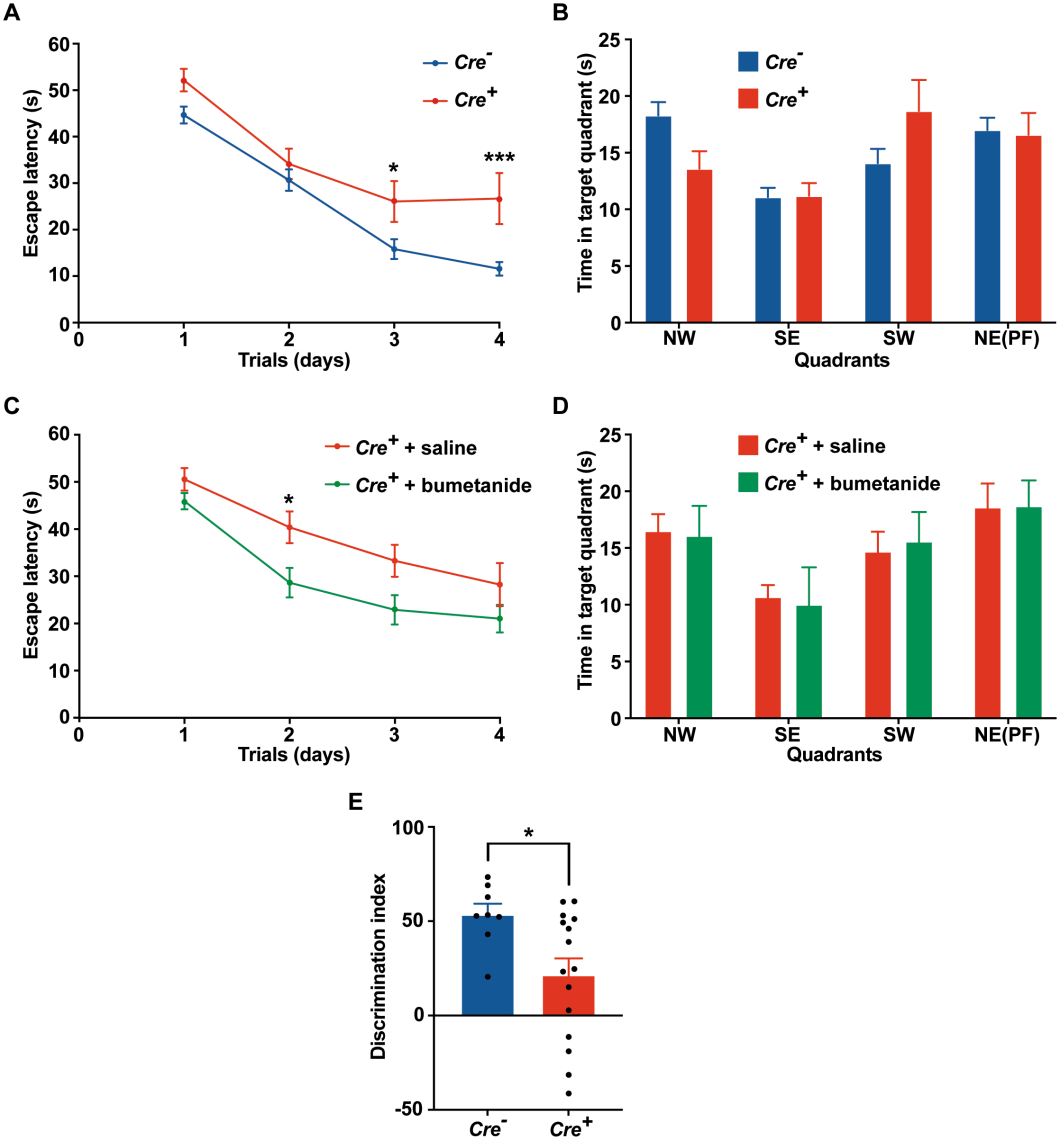
Behavioral evaluation of memory in *Cre^+^* and *Cre-male* mice at 3  months of age. **(A-D)** Morris water maze. **(A)** Escape latency in the MWM compared between *Cre^+^* (*n* = 18) and *Cre^−^* (*n* = 34) mice. **(B)** Probe test: time spent in each quadrant (PF: platform). **(C)** Improvement of escape latency in the MWM after injection of *Cre^+^* mice with saline (*n* = 17) or bumetanide (*n* = 22). **(D)** Probe test: time spent in each quadrant (PF: platform). Values are means ± SEM; ^*^p-value ≤0.05; ^***^p-value ≤0.001; two-way ANOVA with Sidak’s post-hoc test). **(E)** Novel object recognition. Discrimination index in the NOR task (*n* = 8 for *Cre^−^*, *n* = 16 for *Cre^+^* mice; unpaired *t*-test).

**Figure 3:**
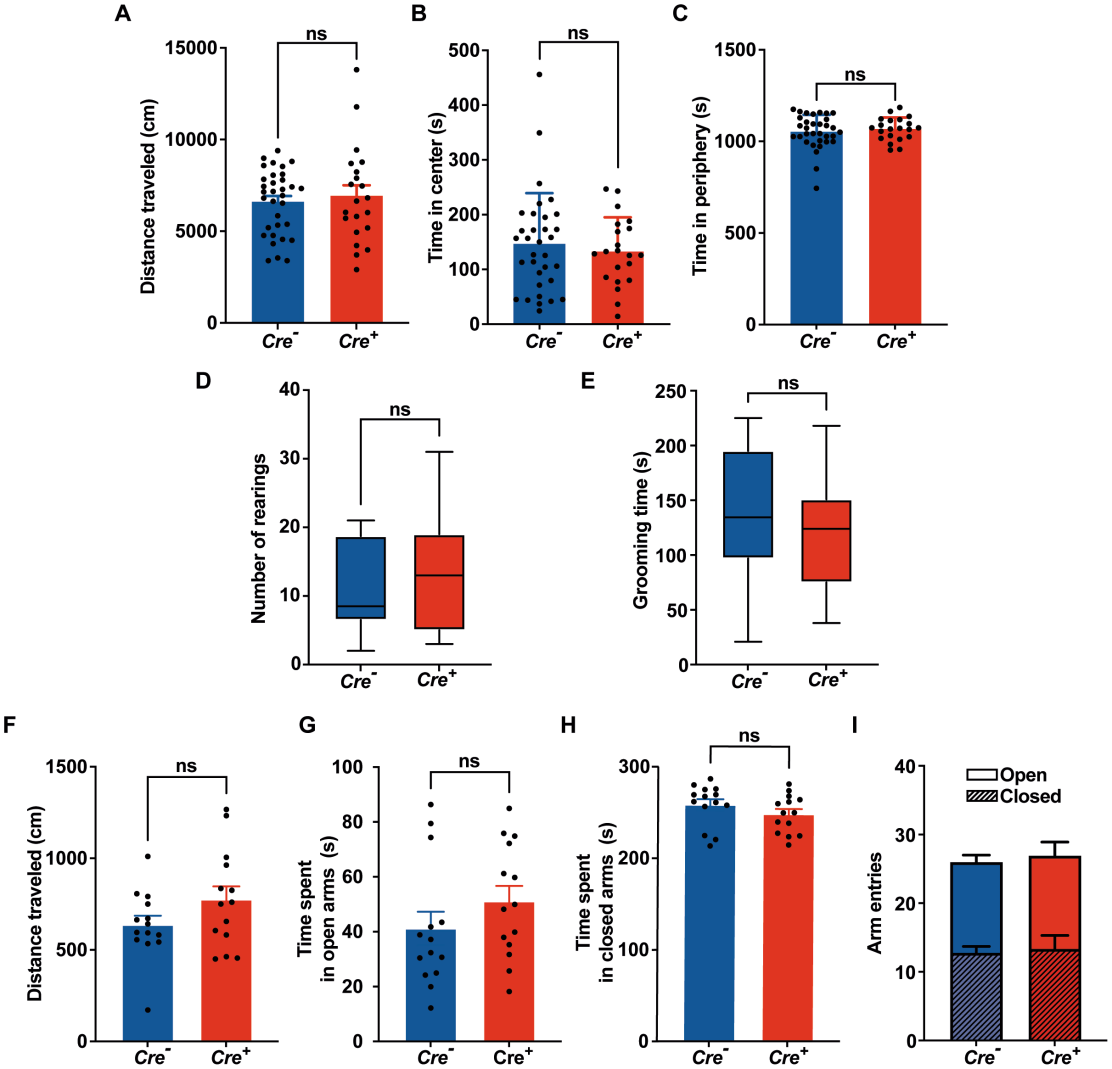
Behavioral evaluation of anxiety. **(A-C)** Open field. **(A)** Distance traveled in the OF. **(B,C)** Time spent in the center and in the periphery of the OF measured in *Cre^−^* (*n* = 33) and *Cre^+^* (*n* = 21) mice (unpaired ***t***-test). **(D)** Number of rearings in 10  min observation (*n* = 8 for *Cre^−^*, *n* = 7 for *Cre^+^*; Mann–Whitney test). **(E)** Total time of grooming bouts observed in 10  min (*n* = 8 for *Cre^−^*, *n* = 7 for *Cre^+^*; Mann–Whitney test). **(F-I)** Elevated plus maze (*n* = 14 for *Cre^−^*, *n* = 14 for *Cre^+^*; unpaired *t*-test). Distance traveled **(F)**, time spent in open **(G)** and closed **(H)** arms, frequency of arm entries **(I)**.

Potential cognitive impairment was analyzed at 3 months of age and after tamoxifen injections in *Cre^+^* and *Cre^−^* mice. The assessment of spatial reference memory was performed using MWM. In the MWM, mice learned the location of a hidden platform with the help of visual cues. The escape latency (time to reach the platform) was measured during the experiment. No apparent locomotor impairment or difference in swimming speed was detected in *Cre^+^* and *Cre^−^* during the MWM trials. On the 1st day (visible platform) and on the 2nd trial day (invisible platform), no significant difference was observed between *Cre^+^* and *Cre^−^* mice. On the 3rd and 4th trial day, *Cre^+^* mice exhibited significantly longer escape latency compared to *Cre^−^* mice indicating an impaired ability of spatial learning due to KCC2 deletion (Figure 2A). However, the probe test performed at the end of the experiment did not show any difference between *Cre^+^* and *Cre^−^* mice suggesting that spatial reference memory could be acquired by *Cre^+^* mice although more slowly than by *Cre^−^* mice (Figure 2B). This experiment was reproduced on *Cre^+^* mice treated with tamoxifen and having been injected intraperitoneally with the selective NKCC1 antagonist bumetanide (0.2 mg/kg 1 h before every test) or saline solution (Figures 2C,D). Bumetanide-treated *Cre^+^* mice reached the platform with a significantly reduced latency, similar to the one observed for *Cre^−^* animals (non-treated with bumetanide). Nonspatial memory was evaluated by the NOR task. This task exploits the natural tendency of rodents to explore novel items and evaluates the ability of the animal to remember an object 24 h after its presentation. We observed that the time spent to explore a new object in comparison to a known object was largely decreased in *Cre^+^* vs. *Cre^−^* mice (Figure 2E). We tried to study the rescue of this impaired memory task by the use of bumetanide; however, we encountered technical problems during the experiments since injected mice (with saline or with bumetanide) were overall very nervous, rendering the test non-conclusive. Exploration behavior as well as locomotor activity was measured in the OF experiment. Deletion of KCC2 had no apparent influence on locomotor activity or exploration behavior in *Cre^+^* mice compared to *Cre^−^* animals (Figures 3A–C). *Cre^+^* and *Cre^−^* mice spent similar time periods in the center area or in the periphery of the OF, suggesting that the decreased expression of KCC2 did not induce anxious phenotype. Rearing and grooming behaviors that are frequently exhibited by mice during fear conditioning were not different in *Cre^−^* and *Cre^+^* mice (Figures 3D,E). In the EPM test used to assess anxiety-related behavior, the preference for being in closed arms over open arms did not differ between *Cre^−^* and *Cre^+^* genotypes, again suggesting that *Cre^+^* mice had no propensity to develop an anxiety phenotype (Figures 3F-I).

Altogether, these results suggest that the decreased expression of KCC2 does not influence locomotor activity but impairs spatial and nonspatial memories and that the performance of *Cre^+^* animals improves after bumetanide treatment.

### KCC2 deletion contributes to intracellular chloride accumulation and a positive shift in E_GABA_

**Figure 4:**
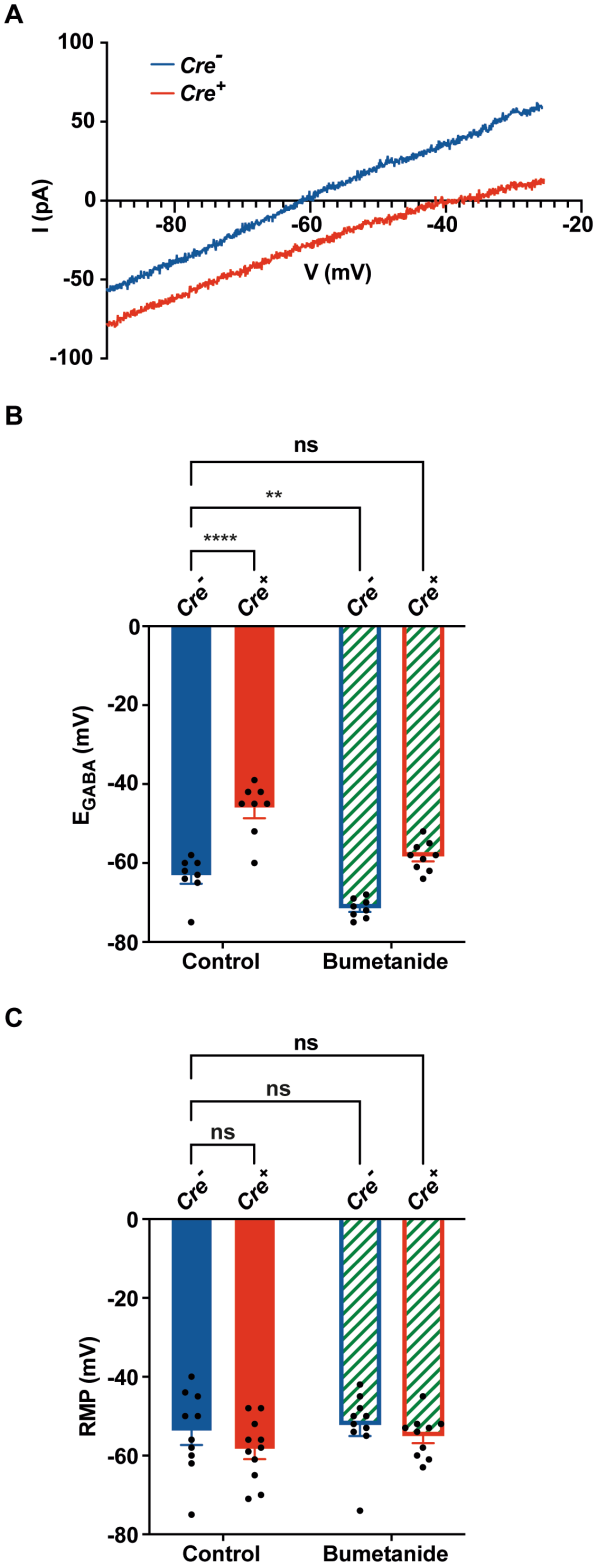
Gramicidin perforated patch clamp experiments on cultured hippocampal primary *Cre^+^* and *Cre^−^* neurons. **(A)** Examples of current–voltage curves of GABA currents elicited by puffing GABA at the neuronal cell body in isolated hippocampal neurons *Cre^−^* (blue) and *Cre^+^* (red). I-V curves are recorded via gramicidin perforated patch clamp and obtained after subtracting the leak current measured at the beginning of each experiment. **(B)** Positive E_GABA_ shift from −63.4 ± 1.9 mV in *Cre^−^* neurons (*n* = 8) to −46.3 ± 2.4 mV in *Cre^+^* neurons (*n* = 8). Bumetanide negatively shifted E_Cl_ of *Cre^+^* neurons back to physiological values (−58.3 ± 1.2 mV, in *Cre^+^* neurons (*n* = 9) and to −71.5 ± 0.9 mV in *Cre^−^* neurons (*n* = 8). Values are mean ± SEM; ^***^p-value ≤0.001, ^**^p-value ≤0.01; two-way ANOVA with the Bonferroni post-hoc test. **(C)** Resting membrane potential values did not statistically differ between of *Cre^−^* neurons (−54 ± 3.3 mV, *n* = 10), *Cre^+^* neurons (−58.5 ± 2.4 mV, *n* = 11), *Cre^−^* neurons treated with bumetanide (−52.3 ± 2.7 mV, *n* = 10), and *Cre^+^* neurons treated with bumetanide (−55.1 ± 1.7 mV, *n* = 10; two-way ANOVA).

To investigate the effect of KCC2 deletion on [Cl^−^]_i_ levels, we measured E_GABA_ on cultured primary hippocampal neurons, using gramicidin perforated patch clamp recordings, a technique that maintains the endogenous intracellular Cl^−^ concentration (Kyrozis and Reichling, 1995). After birth (P0–P1), hippocampi of *Cre^+^* and *Cre^−^* pups were harvested and utilized for primary neuronal cell culture. After 1 week, cells were treated with hydroxy-tamoxifen for 1 week to induce KCC2 deletion. Hippocampal neurons were patched 24 h after the last tamoxifen treatment. Representative current I-V curves of *Cre^+^* and *Cre^−^* hippocampal neurons in the presence of GABA are shown in Figure 4A. We observed that E_GABA_ positively shifted from −63.4 ± 1.9 mV in *Cre^−^* neurons (*n* = 8) to −46.3 ± 2.4 mV in *Cre^+^* neurons (*n* = 8) (Figure 4B), which corresponds to an increase of [Cl^−^]_i_ from 12.9 ± 0.9 mM in *Cre^−^* neurons to 23.6 ± 2.2 mM in *Cre^+^*, confirming the crucial role of the KCC2 transporter in the regulation of intracellular chloride homeostasis through chloride extrusion. As expected, local puffing of GABA in the presence of bumetanide to inhibit the chloride intruder NKCC1 negatively shifted E_GABA_ to −71.5 ± 0.9 mV in *Cre^−^* neurons (*n* = 8) and to −58.3 ± 1.2 mV in *Cre^+^* neurons (n = 9), a value that was close to that observed in untreated *Cre^−^* neurons. The resting membrane potential (RPM), measured in the whole-cell configuration, was similar in *Cre^−^* and *Cre^+^* neurons treated or not with bumetanide (between −52.3 mV and − 58.5 mV, Figure 4C), suggesting that KCC2 ablation does not significantly affect RPM.

### KCC2 deletion induces EPSP-spike potentiation in the hippocampus

**Figure 5:**
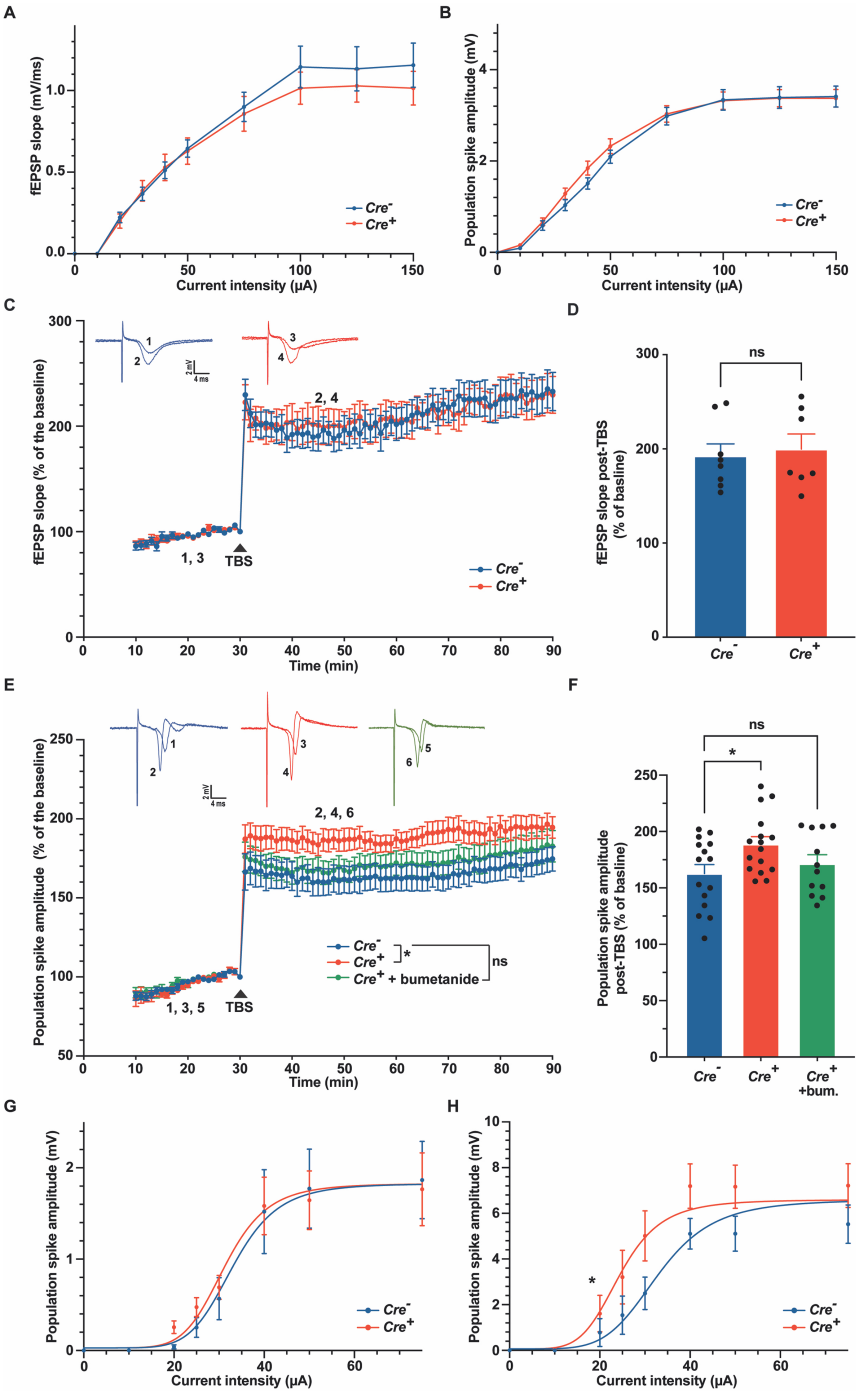
Long-term potentiation in the stratum radiatum and in the stratum pyramidale in *Cre^−^* and *Cre^+^* animals. **(A,B)** Input–output relations measured in the stratum radiatum **(A)**, *n* = 9 for *Cre^−^*, *n* = 7 for *Cre^+^*) and in the stratum pyramidale **(B)**, *n* = 27 for *Cre^−^*, *n* = 44 for *Cre^+^*). (**C,D)**. fEPSP recordings in the stratum radiatum of the CA1 region before and after TBS (slope values normalized to the pre-TBS baseline). Upper insets show representative traces of fEPSP before (1, 3) and 30  min after TBS (2, 4). Time course **(C)** and quantification **(D)** for the period between 15 and 30  min after TBS, i.e., between timepoints 45 and 60  min on panel **C** (*n* = 8 for *Cre^−^*, *n* = 7 for *Cre^+^,* two-way ANOVA (time and genotype) for panel **C**, unpaired *t*-test for panel **(D–F)**. Population spike recordings in the stratum pyramidale CA1 cell layer before and after TBS (amplitude values normalized to the pre-TBS baseline). Upper insets show representative traces of population spikes before (1, 3, 5) and 30  min after TBS (2, 4, 6). Time course **(E)** and quantification **(F)** for the period between 15 and 30  min after TBS (*n* = 20 for *Cre^−^*, *n* = 22 for *Cre^+^*, and *n* = 18 for *Cre^+^* treated with 10  μM bumetanide). ^*^p-value ≤0.05; one-way ANOVA with Dunnett’s multiple comparison test. **(G,H)** Input–output relations measured in the stratum pyramidale 30  min before (*n* = 10 for *Cre^−^* and *n* = 13 for *Cre^+^*) and 30  min after TBS (*n* = 12 for *Cre^−^* and *n* = 13 for *Cre^+^*). Non-linear least squares regression. ^*^p-value ≤0.05; Extra sum-of-squares *F* test.

To further characterize the role of KCC2 in neuronal excitability or synaptic plasticity, we studied LTP in hippocampal slices of *Cre^+^* and *Cre^−^* animals at 3 months of age. The SC were stimulated electrically, and fEPSP and population spikes were recorded in the CA1 hippocampal region, stratum radiatum, and stratum pyramidale, respectively (Figure 5). Input–output curves were similar in *Cre^−^* and *Cre^+^* slices (Figures 5A,B). We chose stimuli consisting of 100 μs pulses of constant currents with intensity adjusted to produce 35% of the maximum response. After 30 min stimulation at 1 stimulation/min, LTP was induced by applying a TBS consisting of five trains of four pulses at 100 Hz with a 200 ms interval between each of the five trains (Wang et al., 2006). fEPSP responses, measured in the CA1 stratum radiatum were normalized to the pre-TBS baseline. As expected, TBS induced a robust LTP to nearly 100% above the basal response that was maintained for at least 1 h. We did not observe any significant difference between *Cre^+^* and *Cre^−^* hippocampal slices (Figures 5C,D). In response to TBS, the population spikes measured in the stratum pyramidale were also potentiated, and interestingly, this potentiation was maintained at a level significantly higher in *Cre^+^* than in *Cre^−^* hippocampal slices (Figures 5E,F). The response observed in control slices (*Cre^−^*) increased a little bit with time so that the difference between *Cre^+^* and *Cre^−^* slices tended to diminish after 1 h. The difference in the population spike activity was not observed anymore when brain slices from *Cre^+^* mice were treated with 10 μM bumetanide (Figures 5E,F). We then repeated measurements of input–output curves in the stratum pyramidale before TBS and 30 min after TBS and observed a significant shift of the curve in *Cre^+^* slices, with an I_50_ value (amplitude of current triggering half of the maximum response) decreasing from 32 μA before TBS to 24 μA after TBS in *Cre^+^* slices, whereas there was no change in *Cre^−^* slices (from 33 μA before TBS to 33 μA after TBS; Figures 5G,H). This demonstrates that the lack of KCC2 reinforces the EPSP-spike potentiation, i.e., an increased ability of EPSPs to generate action potentials.

## Discussion

In the present study, we observed that reducing the forebrain expression of KCC2 and therefore reducing the electrochemical gradient for Cl^−^ induces spatial and nonspatial memory impairments. We used *KCC2^lox/lox^-CaMKIICreERT2^+/−^* mice to reduce the expression of KCC2 at a specific timepoint (at the adult stage thus avoiding developmental problems) and in specific neurons expressing *CaMKIIα*. The highest levels of *CaMKIIα* have been found in the hippocampus (Erondu and Kennedy,1985), with the strongest expression found in the dentate gyrus as well as the pyramidal cell layer of CA1 and CA3 regions (Wang et al., 2013). It is also expressed in many other regions such as in layers II/III and VI of the neocortex, in the thalamus and the hypothalamus, in Purkinje cells of the cerebellum, and in the basal ganglia. Using Tomato reporter gene, we confirmed the expression of *CaMKIIα* in the hippocampus and in the cortex. We also showed that tamoxifen treatment of *KCC2^lox/lox^-CaMKIICreERT2^+/−^* mice largely reduced the expression of KCC2 in the hippocampus, especially in the CA1 region. As expected, neurons isolated from the hippocampus of these mice presented a shift of E_GABA_ to more positive values, reflecting a doubling of [Cl^−^]_i_ inside the cell. This positive shift of E_GABA_ was abolished when cells were treated with the NKCC1 antagonist bumetanide. Increased [Cl^−^]_i_ is expected to reduce the inhibitory action of GABA on GABA_A_R. However, shunting inhibition that relies only on an increase in the postsynaptic membrane conductance should stay intact.

Using MWM and NOR tasks, two tests relying on hippocampus integrity in the conditions used (Terry, 2009; Cohen and Stackman, 2015), we show that *Cre^+^* mice exhibited impairment in both spatial and nonspatial memory compared to control animals. Their exploration abilities were however normal, and we did not observe any anxiety-like behavior. Using hypomorphic KCC2-deficient mice, Tornberg et al. also reported spatial memory alterations and showed a clear anxious phenotype (Tornberg et al., 2005). In the study, we therefore used several tests to study stress-related anxiety-like behaviors like the OF in which the time spent in the center of the arena is taken as a readout for anxiety, or the EPM also based on rodents’ aversion to open spaces (Kraeuter et al., 2019). We did not detect a clear anxious phenotype. In response to anxiety-provoking circumstances, rodents presented a reduction in classical exploratory behaviors such as rearing in which the animal temporarily stands on its hind legs to sample the environment (Sturman et al., 2018). The suppression of exploratory behavior in response to anxiety-provoking conditions is thought to be mediated by hippocampal formation (Lever et al., 2006). We did not observe any reduction in these exploratory behaviors. Self-grooming, a complex innate body care behavior is also classically modified in response to stressful conditions (Kalueff and Tuohimaa, 2005). Again, no differences were observed between *Cre^−^* and *Cre^+^* genotypes. The differences observed between hypomorphic KCC2-deficient mice and our *Cre^+^* mice could stem from developmental problems. We induced KCC2 deficiency in adulthood (3 months of age), while Tornberg et al. presented a model of doubly heterozygous for the KCC2 null and hypomorphic alleles that express 15–20% of normal KCC2 protein level but present during the whole lifespan of the animal, including embryonic and postnatal developmental periods. It has been shown that not only the expression but also the phosphorylation of KCC2 is required for the correct timing of the developmental maturation of Cl^−^-dependent GABAergic neurotransmission and that this process plays a crucial role in the acquisition of adulthood cognitive functions (Moore et al., 2019; Pisella et al., 2019). KCC2 knockout at an early developmental stage has been associated with the decreased neuronal number and increased apoptosis. Cell-specific loss of KCC2 increased apoptotic cell death of projection neurons that were destined for migration into cortical layers. This effect was attributed to the loss of the KCC2 C-terminal domain which plays a role in the ion-transport independent role of KCC2. The C-terminal end of KCC2 is able to control downstream actin dynamics by regulating cofilin phosphorylation, a major actin-regulation protein (Mavrovic et al., 2020). Alternatively, the difference between hypomorphic KCC2-deficient mice and our *Cre^+^* mice might be due to the more restrained localization of our KCC2 knockdown, limited to neurons expressing *CaMKIIα*, i.e., the hippocampus, several regions of the neocortex, the thalamus, the hypothalamus, the Purkinje cells of the cerebellum, and the basal ganglia. Noteworthy, hypomorphic KCC2 weighs 20% less compared to wild-type littermates (Tornberg et al., 2005). Although we did not notice any weight changes in *Cre^+^* mice since the KCC2 deletion occurred after birth and when mice already had reached adulthood, we nevertheless observed in the first experiment that several *Cre^+^* mice died after 4 or 5 weeks post-tamoxifen treatment, presumably after epileptic seizures. We therefore chose to complete experiments during the 2nd and 3rd week after tamoxifen treatment, and mice were euthanized at the end of the 3rd week post-tamoxifen treatment.

Impairment in spatial reference memory was rescued after treatment of the animals with bumetanide suggesting that behavioral effects observed in *Cre^+^* mice were indeed due to impaired Cl^−^ regulation. However, caution must be taken in the interpretation of this *in vivo* observation. Indeed, it has been shown that at the low clinical doses approved for diuresis, bumetanide has only weak access into the CNS, reaching levels that are below the concentration required to inhibit NKCC1 (reviewed in (Loscher and Kaila, 2022)). On the contrary, a series of laboratories have observed anticonvulsant effects of bumetanide in several seizure models and in different pathological states. At the dose used in the present study (IP injection of 0.2 mg/kg), Sivakumaran and Macguire presented data that highly suggest a central action of the drug in a model of kainic acid-induced status epilepticus (Sivakumaran and Maguire, 2016). These authors and others propose that seizures (also observed in our mouse model) cause a breakdown in the blood–brain barrier, which may facilitate the central actions of bumetanide (Pont et al., 1995). However, at this stage, it cannot be excluded that bumetanide exerts its action via targets other than NKCC1 and/or outside of the CNS. For this reason, several strategies are currently used to develop new drugs or prodrugs of bumetanide that ameliorate the blood–brain barrier passage (Tollner et al., 2014; Savardi et al., 2020).

The role of KCC2 in synaptic plasticity is not well known and has not been studied in conditional knock-out models so far. Ferrini et al. found that low KCC2 expression enhances long-term facilitation measured by field potentials recordings in the spinal cord (Ferrini et al., 2020). However, Chevy et al. showed that the downregulation of KCC2 precludes LTP in the perforant pathway of the dentate gyrus (Chevy et al., 2015). LTP of CA3-CA1 glutamatergic transmission has been the most studied model of synaptic plasticity probably because it has been shown to be required *in vivo* for hippocampal-dependent spatial and temporal learning (Morris et al., 1986; Tsien et al., 1996; Huerta et al., 2000). The firing of presynaptic CA3 pyramidal neurons produces monosynaptic EPSPs followed rapidly by feedforward disynaptic inhibitory postsynaptic potentials (IPSPs) originating from basket cells that target the somatic compartment (Pouille and Scanziani, 2001; Glickfeld and Scanziani, 2006). Ormond and Woodin showed that feedforward inhibition reduces EPSP amplitude recorded at the soma and that disinhibition at feedforward synapses expressed as a depolarization of the reversal potential for GABA_A_R-mediated currents contributes to the increase in EPSP amplitude seen during LTP expression (Ormond and Woodin, 2009, 2011). In the present study, we observed that *Cre^+^* and *Cre^−^* mice exhibited a similar potentiation of fEPSP in the stratum radiatum of the CA1 region after TBS. However, population spikes measured in the somatic layer were significantly more potentiated by TBS in *Cre^+^* than in *Cre^−^* brains. The observed shift of the input–output relation to a lower I_50_ value in *Cre^+^* brain slices after TBS stimulation indicated a reinforcement of the EPSP-spike potentiation, i.e., an increased ability of EPSPs to generate action potentials. This effect could thus be explained by a reduced feedforward inhibition of somatic response in the absence of KCC2 expression.

Wang et al. have shown that TBS-induced LTP is accompanied by a progressive diminution of KCC2 expression (typically 30% decrease 1 h after TBS) (Wang et al., 2006). This means that TBS potentiates EPSPs, and at the same time, it reduces the activity of KCC2, which facilitates EPSP-induced action potentials. In this study, having a reduced expression of KCC2, *Cre^+^* mice exhibited more potentiated population spikes. The progressive reduction of KCC2 expression in control mice probably explains why the difference observed between *Cre^+^* and *Cre^−^* mice tends to diminish with time. Therefore, our results show that downregulation of KCC2 is not required for induction and maintenance of LTP but supports the fact that such downregulation induces EPSP-PS facilitation.

The fact that TBS-induced LTP is increased whereas spatial memory is impaired may appear surprising as LTP is classically considered as the cellular mechanism that undergoes experience-dependent changes in synaptic connections and information storage. However, such apparent dissociation between LTP and memory has been observed in mice expressing mutant postsynaptic density protein-95 (Migaud et al., 1998) in mice with forebrain-specific ablation of *Stim* genes (Garcia-Alvarez et al., 2015) or in mice overexpressing the amyloid precursor protein APP that exhibit alterations in GABA neurotransmission (Kreis et al., 2021). To explain this effect, Pineda et al. hypothesized that amplified responsiveness of CA1 postsynaptic neurons to stimuli would saturate LTP and diminish synapse-specific plasticity required for new memory formation (Pineda et al., 2004). Interestingly, Ferando et al. reported that KCC2 levels were decreased in old animals and that LTP extended to unstimulated synapses, thereby weakening its synaptic specificity (Ferando et al., 2016). Memory impairments during senescence could therefore not necessarily result from deficits in LTP induction, but possibly result from the spread of potentiation to unstimulated synapses.

Altogether, our results thus show a change in the expression of KCC2 with its inherent consequences on [Cl^−^]_i_ and GABA signaling, alters synaptic plasticity, and modulates spatial and nonspatial memory. It also indicates that KCC2 and NKCC1 are potential targets in the treatment of cognitive disorders.

## Data availability statement

The raw data supporting the conclusions of this article will be made available by the authors, without undue reservation.

## Ethics statement

The animal study was reviewed and approved by Animal Ethics Committee of the Université catholique de Louvain (2016/UCL/MD/026 and 2020/UCL/MD/015). Written informed consent was obtained from the owners for the participation of their animals in this study.

## Author contributions

PG conceived the study. RG, J-NO, NP, and PG designed the experiments. AK, FI, XY, CJ, OS, MC, and RG performed the experiments. AK, FI, XY, CJ, OS, NT, and PG analyzed the data. AK and PG wrote the manuscript text. All authors contributed to the article and approved the submitted version.

## Funding

This study was supported by the Queen Elisabeth Medical Foundation, the Concerted Research Action from the General Direction of Scientific Research of the French Community of Belgium (ARC17/22–083), and the Belgian Fund for Scientific Research (FNRS, grant PDR -T.0089.2).

## Conflict of interest

The authors declare that the research was conducted in the absence of any commercial or financial relationships that could be construed as a potential conflict of interest.

## Publisher’s note

All claims expressed in this article are solely those of the authors and do not necessarily represent those of their affiliated organizations, or those of the publisher, the editors and the reviewers. Any product that may be evaluated in this article, or claim that may be made by its manufacturer, is not guaranteed or endorsed by the publisher.
